# Editorial: Continuous glucose monitoring: beyond diabetes management

**DOI:** 10.3389/fendo.2025.1678600

**Published:** 2025-08-21

**Authors:** Jianzhong Xiao

**Affiliations:** Department of Endocrinology and Metabolism, Beijing Tsinghua Changgung Hospital, Tsinghua University Clinical Medical School, Tsinghua University, Beijing, China

**Keywords:** continuous glucose monitoring, diabetes, diabetes management, personalized nutrition, glycemic variability, metabolic health

Since the establishment of the causal relationship between blood glucose levels and diabetes complications, glycemic control has become a cornerstone of diabetes metabolic management (1). In recent years, continuous glucose monitoring (CGM) systems have emerged as transformative tools for diabetes care (2). By measuring glucose levels in interstitial fluid, CGMs provide near-continuous real-time glucose readings and comprehensive ambulatory glucose profiles (AGP) (3). These capabilities are critical for optimizing insulin dosing, dietary planning, and physical activity management (4–6). The real-time visualization of glycemic variability has not only revolutionized diabetes treatment but also significantly enhanced patients’ quality of life (7). Moreover, CGM-derived metrics such as Time in Range (TIR), Time Below Range (TBR), and Time Above Range (TAR) have introduced new paradigms in glycemic assessment (3). Notably, the application of CGM is expanding beyond traditional diabetes management, opening new frontiers in personalized health optimization. This article reviews current evidence including articles in this Research Topic, to discuss the potential applications, limitations, and prospects of CGM technology.

## Beyond diabetes management: expanding applications of CGM

We know that glucose is a major substrate of energy metabolism and a core player in overall metabolic health, energy regulation, and cellular function. Fluctuations, even within physiologically “normal” ranges - can profoundly influence wellbeing and performance (8). This understanding has driven growing interest in CGM applications among non-diabetic populations:

1. Optimize metabolic health & prevent diabetes:

CGM provides immediate, personalized feedback on how specific foods and dietary components (carbohydrate type/fiber/fat/protein ratio, serving size, order) affect blood glucose (9). One person’s “healthy” meal can cause another person’s blood sugar to spike dramatically (10). CGM provides support for truly personalized dietary choices to minimize harmful blood sugar spikes and promote metabolic stability.

2. Early identification and prevention of dysglycemia: CGM can reveal abnormal blood glucose fluctuations long before standard fasting blood glucose or HbA1c tests show abnormalities (11). Users seeing frequent or prolonged postprandial spikes can be a powerful motivator for lifestyle interventions to prevent the progression to type 2 diabetes (12).

3. Athlete nutrition management and training intensity monitoring (13).

Athletes rely heavily on glycogen stores. CGM helps them understand how different fuel strategies affect glucose availability and stability during training and competition. At the same time, glycemic patterns after exercise can provide clues about recovery status and the effectiveness of energy replenishment. A stable blood glucose overnight after strenuous activity indicates adequate nutrition, while an unstable blood sugar may indicate inadequate intake or constant stress. CGM can sometimes show how different training loads or types affect glucose regulation, potentially marking over-training states.

4. Weight Management and Body Composition Goals:

The “calorie intake, calorie burn” model is increasingly seen as oversimplifying. A spike in blood sugar triggers the release of insulin, a hormone that promotes fat storage and can inhibit fat burning (14). To identify foods that cause significant spikes and to choose dietary variety(i.e. low carbohydrate high protein) promoting more stable glucose and insulin levels, potentially create a more favorable hormonal environment for fat loss and muscle gain (15). Furthermore, large blood glucose spikes are often accompanied by rapid drops, which can trigger hunger, fatigue, and cravings – especially for more carbohydrates or sugar (16). Minimizing these spikes through dietary modification probably supports adherence to healthy eating patterns.

5. Understand energy, mood, and nerve function:

Many people with diabetes experience depression. CGM can directly link these subjective feelings to blood sugar levels (17). Minimizing extreme blood sugar fluctuations by taking a CGM may help some people improve the nerve response to hypoglycemia (18).

6. Women’s Health and Hormonal Fluctuations:

Hormones such as estrogen and progesterone significantly affect insulin sensitivity. In most of the study population, glucose levels rose linearly throughout the menstrual cycle, reaching a maximum in the late luteal phase. A sharp decrease was seen in women at the beginning of menstrual bleeding (19). Polycystic ovary syndrome (PCOS) is often associated with insulin resistance. CGM can be a valuable tool for managing blood glucose levels in women with PCOS.

7. Longevity and diets intervention:

Some researchers hypothesize that minimizing high blood sugar spikes and excessive variability (even within normal limits) may reduce oxidative stress and inflammation, potentially slowing the aging process (20). CGM provides data to proactively manage this variability. Biohackers used CGM to test the effects of various interventions—specific diets (ketosis, intermittent fasting), supplements, sleep patterns, stress reduction techniques—on their blood glucose profile, seeking optimal metabolic function (21).

8. Enhancing Quality of Life in Special Diabetic Populations.

Type 1 diabetes management faces unique challenges during Ramadan, where patients experience dawn phenomenon before Suhoor meals, post-Iftar hyperglycemia, and nocturnal hypoglycemia risks. (Alguwaihes et al.) Similarly, pregnant women with diabetes endure significant psychological burdens from stringent glycemic targets. Liu et al. found CGM improved self-rating anxiety, pregnancy-related anxiety, and diabetes specific quality of life. While advanced technologies like sensor-augmented pumps with automated insulin suspension theoretically alleviate hypoglycemia fear syndrome (HFS), current evidence indicates limited improvement regardless of SmartGuard™ or CGM implementation—potentially due to insufficient usage duration. (Schierloh et al.) Notably, CGM offers unique advantages for evaluating novel hypoglycemic agents through comprehensive pharmacodynamic profiling of glucose excursions, surpassing traditional spot-check measurements (Wei et al.).

## Technical limitations and future trajectory

Despite promising applications, CGM technology faces inherent physiological constraints. Accuracy needs next generation technology or systemic calibration. (Wu et al.) The 5–15 minute physiological lag between interstitial fluid and blood glucose measurements becomes particularly problematic during rapid glycemic fluctuation (22). Accuracy challenges persist during intense physical activity and other metabolic stressors (Maytham et al.). Implementation barriers include clinical data overload (“glucose fatigue”), reimbursement limitations for non-diabetic applications, and privacy concerns regarding cloud-stored health data (23). Future developments will likely focus on multimodal biometric integration, machine learning-enhanced predictive alert systems, and closed-loop systems for health optimization. These innovations may ultimately transform CGM from a monitoring tool into integrated health management platforms (24).
